# HIV and alcohol knowledge, self-perceived risk for HIV, and risky sexual behavior among young HIV-negative men identified as harmful or hazardous drinkers in Katutura, Namibia

**DOI:** 10.1186/s12889-015-2516-5

**Published:** 2015-11-26

**Authors:** Amee Schwitters, Jennifer Sabatier, Puja Seth, Mary Glenshaw, Dietrich Remmert, Sonal Pathak, Naomi Bock

**Affiliations:** Division of Global HIV/AIDS, Center for Global Health, Centers for Disease Control and Prevention, 1600 Clifton Road NE, Mailstop E-30, Atlanta, GA 30329-1902 USA; Division of Global HIV/AIDS, Center for Global Health, Centers for Disease Control and Prevention, Windhoek, Namibia; ICF International, Atlanta, GA USA

**Keywords:** Namibia, Alcohol, HIV, Men

## Abstract

**Background:**

Namibia’s HIV prevalence is 13.3 %. Alcohol is associated with sexual risk-taking, leading to increased HIV risk. Baseline sexual behaviors, HIV and alcohol knowledge, and self-perceived HIV risk were examined among men reporting high-risk drinking in Katutura, Namibia.

**Methods:**

HIV negative men, ≥ 18 years, were screened for harmful or hazardous levels of drinking and >1 recent sex partner prior to randomization into control or intervention arm. SAS 9.3 and R 3.01 were used for descriptive baseline cohort analyses.

**Results:**

A total of 501 participants who met criteria were included in analysis (mean Alcohol Use Disorders Identification Test [AUDIT] =12.4). HIV and alcohol knowledge were high with the majority (>85 and 89.8–98 %, respectively) of respondents correctly answering assessment questions. Despite high knowledge levels, 66.7 % of men felt they were at some or high risk of HIV acquisition. Among those respondents, 56.5 % stated often wanting to have sex after drinking and 40.3 % stated sex was better when drunk. Among respondents with non-steady partners [*n* = 188], 44.1 % of last sexual encounters occurred while the participant was drunk and condoms were not used 32.5 % of those times. Among persons who were not drunk condoms were not used 13.3 % of those times.

**Conclusions:**

Sex with casual partners was high. Inconsistent condom use and alcohol use before sex were frequently reported. Increased emphasis on alcohol risk-reduction strategies, including drinking due to peer pressure and unsafe sexual behaviors, is needed.

## Background

Despite representing only 12 % of the world’s population, sub-Saharan Africa continues to carry a disproportionate burden of HIV, with 70 % of all global HIV cases residing in this region [1]. Located in sub-Saharan Africa, Namibia has one of the highest HIV adult prevalences in the world, with an estimated adult prevalence of 13.3 % [1].

Alcohol use has a well-established association with sexual risk-taking that can lead to HIV acquisition and transmission [2]. Research has repeatedly shown that alcohol use is related to sexual risk-taking behavior, including increased number of sexual partners, inconsistent condom use, and increased incidence of sexually transmitted infections (STIs) [3–10]. Alcohol use can reduce the ability to learn and to perform sexual risk-reduction strategies [11]. Beliefs about the effects of alcohol use on sexual activity and the perceived degree of alcohol intoxication moderate both the motivations to engage in safer sex and the behavioral skills needed to negotiate condom use with a sexual partner [12].

Alcohol drinkers engage in more sexual risk behaviors compared to non-drinkers, including higher number of sexual partners, more sex with casual partners, and decreased frequency of condom use, along with a higher chance of improper condom use [11, 13–16]. High quantities of alcohol intake during a single occasion (binge drinking, which is typically defined as five or more drinks in a sitting for men) and frequent drinking are both associated with higher prevalence of HIV infection in cross-sectional surveys. Also, prevalence for HIV and other STIs is higher among drinkers compared to non-drinkers [17]. In addition, drinking establishments have been reported to be venues to meet casual and transactional sex partners, further contributing to HIV acquisition and transmission risk [3].

Several studies from southern Africa have demonstrated the association between alcohol use and HIV acquisition and transmission, unprotected sex, multiple sex partners, and STI incidence [3–5, 14, 18–20]. Within southern Africa, alcohol use is typically characterized by harmful patterns such as high quantities of alcohol consumption per occasion, drinking in public spaces, heavy alcohol use during cultural events, and drinking outside mealtimes [4, 5, 21, 22]. In the region, Namibia and South Africa have the highest documented consumption levels of alcohol [23]. In Namibia, per capita consumption of alcohol among persons 15 years and older has increased between 1961 and 2005, with beer being the most commonly consumed alcoholic beverage.

Studies dating back to the mid-1990s have shown high levels of alcohol consumption in Namibia with usage generally higher among men [24] although a report showed indications of decreasing trends in use [25]. In 2002, the Namibian Ministry of Health and Social Services (MoHSS) found that 56 % of Namibian adults were current drinkers and that drinking rates in the capitol, Windhoek, were 70 %. Among those who drank, 45 % in Windhoek reported recent binge drinking (defined in this setting as more than 6 units of alcohol per occasion) and 27 % reported the need to consume alcohol in the morning [26]. In the 2011 World Health Organization (WHO) Global Status Report on Alcohol and Health, Namibia was given a moderate score of three on a five-point scale assessing alcohol-attributable burden of disease [23].

The purpose of this manuscript is to present baseline data of study participants examining knowledge about HIV and alcohol, self-perceived risk for HIV, the number of sex acts preceded by alcohol use, and the number of sex acts using condoms among HIV-negative men who were seeking services at a voluntary HIV counseling and testing (VCT) site in Katutura, Namibia.

## Methods

### Location

The specific VCT site utilized in this study was chosen because it is the largest in Katutura and is well known. As of August 2010, the site reported an average of 45 clients receiving VCT services at the center each day, six days a week, and 50 % were male. Katutura is a large former township on the outskirts of the Namibian capitol, Windhoek. Katutura was established for non-white residents prior to independence during the apartheid era, however it now includes an ethnically diverse population of approximately 100,000–200,000 persons.

### Participants

To be eligible to participate in the randomized controlled trial of the behavioral counseling intervention, participants had to be men at least 18 years of age who tested HIV-negative at a VCT site in Katutura, Namibia. Additional eligibility criteria included self-report of harmful or hazardous alcohol consumption and more than one sexual partner in the previous three months. Hazardous or harmful drinking was determined as having a Alcohol Use Disorders Identification Test (AUDIT) score of eight to 19 (WHO defined risk levels of harmful or hazardous drinking) [26]. Men had to be able to provide informed consent, have access to a cell phone, have plans to stay in the area for six months, after the beginning of the trial, and be able to communicate verbally in English, Oshiwambo, or Afrikaans.

### Study methods

Study recruitment and follow up took place from February 2011 to April 2013. Men were approached consecutively as they entered the VCT and asked if they would be willing to participate in the study. Men were initially screened for eligibility based on age, language, residency, alcohol consumption in the last month, HIV status, HIV test day of screening, sexual partners in the last three months, possession of cell phone (eligibility requirements described in more detail above). Men who passed the initial screening were then screened for alcohol use using the AUDIT questions. Men who also passed the alcohol screening were then given the consent form to review with screener.

A total of 8004 men were approached about the study; 7163 were screened (89.5 %) and 1243 (17.4 %) were found eligible. After a second round of eligibility screening, a total of 573 (46.1 %) men were eligible to participate in the study based on the criteria explained above. Written informed consent was obtained from the participants prior to beginning data collection. The protocol was reviewed by the Namibian Ministry of Health and Social Services Ethics Review Board and The Centers for Disease Control Institutional Review Board and received final approval in December 2010. Due to administrator error and miscommunication, the study was not registered at clinicaltrials.gov until after study completion, although the study protocol was extensively reviewed by both boards/committees prior to implementation. The present study includes all men in the randomized controlled trial and presents baseline assessment data. For additional details on intervention methodology, see clinicaltrials.gov.

All participants were given a study ID card with the dates for the three- and six-month follow up appointments. After completion of the baseline assessment men received a $7.00USD (N$50) grocery store voucher for their time. After each follow up appointment, men were given taxi fare ($2.00USD) and a $7.00USD grocery store voucher for the time.

### Measures

#### Sociodemographic and access to healthcare characteristics

Participants were asked about their current age, residential location, educational attainment, employment status, marital status, children, and socioeconomic-related questions.

#### Self-risk perception and exposure to HIV intervention and HIV knowledge

Participants were asked if a health care worker had ever talked with them about their sex behaviors, their alcohol use or the connection between alcohol use and riskier sex. The survey also assessed self-perceived HIV risk, condom use during sexual encounters, and alcohol use before sexual encounters. Questions were asked about HIV knowledge, including routes of transmission and treatment.

#### Alcohol use and alcohol-related knowledge

Alcohol-related knowledge included questions on physiological effects of heavy alcohol use, impact of alcohol use on sexual function and ability to practice safe sex, coping with daily stressors, and addiction. The AUDIT questionnaire, where total scores range from 0 to 40, [27] was administered to determine eligibility. Men were categorized into 3 drinking categories: non-drinkers (AUDIT = 0), non-harmful drinkers (AUDIT = 1–7), and harmful/likely dependent drinkers (AUDIT ≥ 8). As mentioned previously, men were eligible to participate, if they had scores from 8 to 19. In addition to the AUDIT, men were asked about types of alcohol consumed. The 4-item CAGE alcohol screen also was embedded in the AUDIT screen. The CAGE has been shown to be an accurate method of screening for problematic alcohol usage patterns and a score of two or more is defined as being clinically significant and an indication of alcohol problems [28]. The results of the CAGE and AUDIT screens were evaluated to determine if they identified the same men as harmful or hazardous drinkers.

#### Condom use and sexual behavior history

Condom use, including frequency of use, partners with whom condoms were used, and reasons for not using condoms were assessed. Sexual activity questions included a detailed history for up to four sexual partners in the previous six months. Data collected included partner sex, partner type, frequency of sexual activity, HIV and pregnancy prevention, alcohol use related to sexual activity, including if the partner was initially met at a drinking venue. Knowledge of partner HIV status and testing history were also assessed.

### Data analysis plan

Performed univariate analyses included frequencies, percentages, means, and standard errors, where applicable. The analyses were conducted using SAS 9.3 and R 3.01.

## Results

Of the 573 men eligible to participate in the study, 572 were included (99.8 %) and 550 (96.2 %) consented to participate in the study. Of those providing consent, 501 (91.1 %) completed baseline data collection (87.4 % of the 572 included men). The mean age of participants was 26.8 years (Table 1). The mean AUDIT score was 12.5 (95 % confidence interval [CI] = 12.18–12.71) and indicative of harmful alcohol use. Beer was the most frequently consumed [62.5 %; 95 % CI = 57.63–66.16] alcoholic beverage, with a daily average of 4.7 [95 % CI = 4.46–4.96] alcoholic beverages consumed. Men included in this study were identified as engaging in harmful or hazardous alcohol use, as defined by the AUDIT. Among them, 90.2 % [*n* = 452] of them also had CAGE scores of two or greater (identified as clinically significant for the identification of problem drinking) [26].

**Table 1:** Baseline characteristics of respondents, Katutura, Namibia, 2011–2013 (*n* = 501)

Characteristic	*n*	%	95 % CI
Marital status			
Dating/not living with	320	63.9	59.66–68.08
Dating/living with	89	17.8	14.41–21.12
No partner	66	13.2	10.21–16.14
Married	26	5.2	3.24–7.13
Education level			
Secondary	417	83.2	79.96–86.50
Higher	58	11.6	8.77–14.38
Primary	21	4.2	2.43–5.95
Other/Do not know	5	1.0	0.13–1.87
Main occupation			
Employed, formal sector	218	43.5	39.17–47.86
Looking for work	118	23.6	19.83–27.27
Student	101	20.2	16.64–23.68
Employed, informal sector	53	10.6	7.88–13.28
Other	11	2.2	0.91–3.48
Water source			
House	429	85.6	82.55–88.70
Borehole (well water)	72	14.4	11.30–17.45
Electricity in house			
Yes	433	86.4	83.42–89.43
No	68	13.6	10.57–16.58
Type of Toilet			
Flush	446	89.0	86.28–91.76
No facility	40	8.0	5.61–10.36
Ventilated pit latrine	11	2.2	0.91–3.48
Pit latrine	4	0.8	0.02–1.58
Rooms used for sleeping in house			
Three or more	321	64.0	58.86–68.28
Two	118	23.6	19.83–27.27
One	62	12.4	9.49–15.26
	Mean		
Age	26.8		26.34–27.47
Number of children supported	1.8		1.63–1.96
AUDIT Score	12.4		12.18–12.71
Average number of alcoholic drinks per day	4.7		4.46–4.96

Among participants, 63.9 % [95 % CI = 59.66–68.08] reported dating but not living with a partner and 37.5 % [95 % CI = 59.66–68.08] reported their primary partner to be casual, a friend, paid, or other, which was classified as “non-steady” partner. The mean number of sex partners in the last six months was 3.0 [95 % CI = 2.78–3.12]. Among respondents, 40.3 % [95 % CI = 36.08–44.62] stated sex was better when they were drunk and 56.5 % [95 % CI = 52.14–60.83] stated they often wanted to have sex after drinking (Table 2). Among men with non-steady partners [*n* = 188], 44.1 % (95 % CI: 37.0–51.3) of last sexual encounters occurred while the participant was drunk and condoms were not used 32.5 % (95 % CI: 22.2–42.8) of those times. Among persons who were not drunk, condoms were not used 13.3 % (95 % CI: 6.8–19.9) of those times.

**Table 2:** Sexual risk factors, Katutura, Namibia, 2011–2013 (*n* = 501)

	N	%	95 % CI
Number partners in the last six months	3.0 (mean)	---	2.78–3.12
Want sex after drinking			
Agree	283	56.5	52.14–60.83
Disagree	218	43.5	39.16–47.86
Sex is better when drunk			
Disagree	296	59.1	54.77–63.39
Agree	202	40.3	36.08–44.62
Do not know	3	0.6	0–1.28
Self-perceived risk for HIV			
Some-risk	179	35.7	31.53–39.93
High-risk	155	30.9	26.88–34.99
Little-risk	122	24.4	20.59–28.12
No-risk	44	8.8	6.30–11.26
Do not know	1	0.2	0–0.59
Self-perceived risk for HIV related to alcohol use			
Yes	337	67.3	63.15–71.38
No	162	32.3	28.23–36.44
Do not know	2	0.4	0–0.95

Less than half of participants reported that health care workers had ever talked to them about their alcohol use [23.2 %; 95 % CI = 19.45–26.85], their sexual activity [36.1 %; 95 % CI = 31.91–40.34] or their alcohol use and association with riskier sex [39.3 %; 95 % CI = 35.04–43.60], but knowledge of the health effects of alcohol was high. Most men correctly identified that heavy alcohol use can lead to brain [96.2 %; 95 % CI = 94.53–97.88] and liver damage [98.0 %; 95 % CI = 96.78–99.23], decrease the effectiveness of the immune system [89.8 %; 95 % CI = 87.17–92.47], and that alcohol is addictive [96.2 %; 95 % CI = 94.53–97.88].

HIV knowledge among participants was also high, with over 85 % correctly answering the following statements: using condoms correctly reduces risk of HIV [98.4 %; 95 % CI = 97.30–99.50]; having many sexual partners increases HIV risk [97.8 %, 95 % CI = 96.52–99.09]; HIV is not caused by witchcraft [96.2 %; 95 % CI = 94.53–97.88]; a healthy looking person can have HIV [95.4 %; 95 % CI = 93.57–97.24]; and a person can have HIV and not know it [95.2 %; 95 % CI = 93.34–97.08].

Participants who reported not always using a condom cited the following as main reasons for non-use: not having condoms [25.1 %; 95 % CI = 21.35–28.95], feeling trust for their sexual partner [24.0 %; 95 % CI = 20.21–27.70], reporting that sexual partner was pregnant [15.4 %; 95 % CI = 12.39–18.75], reporting inability to use condoms when drunk [14.6 %; 95 % CI = 11.48–17.66], reporting forgetting to use condoms when drunk [11.6 %; 95 % CI = 8.42–13.94], reporting ignorance of where to get condoms [6.6 %; 95 % CI = 4.41–8.76], reporting love for their sexual partner [5.6 %; 95 % CI = 3.58–7.60], and reporting that sex is better without condoms [4.6 %; 95 % CI = 2.76–6.43].

Finally, men were asked to think about their own behaviors and rate their self-perceived HIV risk. Despite their high levels of HIV transmission and prevention knowledge, 66.7 % [95 % CI = 62.53–70.80] stated they felt that were at some or high risk of HIV acquisition and 67 % [95 % CI = 63.15–71.38] felt their alcohol use was related to their risk for contracting HIV. Sixty-five percent [95 % CI = 60.89–69.25] of men stated they feel pressured to drink by friends, 55.5 % [95 % CI = 51.13–59.85] had been in a physical fight while drunk, and 28.3 % [95 % CI = 24.39–32.30] had hit their female partner or another women while drunk.

## Discussion

This study examined HIV and alcohol knowledge, self-perceived risk for contracting HIV, and sexual behavior among HIV-negative men seeking VCT services in Katutura, Namibia. Despite the high HIV prevalence and high rates of alcohol consumption in Namibia, a limited number of studies have been conducted to better understand these psychosocial factors and behaviors among this population. This sample of men reported very high levels of knowledge about sexual risk behavior, HIV, and harmful consequences related to high levels of alcohol consumption, yet still perceived themselves to be at risk for HIV and reported harmful to hazardous levels of alcohol use. Over one-third of participants stated their primary sexual partners were non-steady partners. Inconsistent condom use and alcohol use before sex were also reported. Sex with multiple partners and/or casual partners has been associated with frequent and heavy alcohol consumption in prior studies [29–31]. These findings support the fact that knowledge is not sufficient to change risky behavior [32]. Therefore, other factors, such as self-efficacy, skill-level, and other contextual and structural-level factors may be impacting high levels of alcohol use and risky sexual behaviors.

Two-thirds of the men reported they felt pressured by their peers to drink. Similar to previous findings from Namibia, beer was the most commonly consumed beverage, perhaps due to it being readily available and affordable in most countries [23]. Within this sample, 14.5 and 11.5 % of men stated they did not use condoms when drunk or forgot to use condoms when they were drunk, respectively. With multiple casual partners, this presents an opportunity for long-term consequences, such as HIV or STI acquisition. This study adds to prior literature demonstrating a relationship between alcohol consumption and adverse health outcomes [33–35].

Given the large number of men indicating the impact of peer influence on drinking behaviors, positive peer-based alcohol use reduction strategies, along with lessons on healthy sexual behaviors, may reduce HIV risk among this group. Peer-based education has been used successfully in Chennai, India to reduce alcohol use and risky sexual behavior through the incorporation of risk-reduction messaging, support for peer educators, and an interactive curriculum [36].

This study has limitations including those common to studies relying on self-reported data. Social desirability bias may have influenced some participants to underreport their alcohol use or overreport their condom use. Recall bias may have impacted participants’ responses when asked about previous alcohol use and risky sexual behavior. Additionally, the study was conducted in an urban area and with men planning to stay in the same area for six months after initiation into the study. Alcohol use and sexual risk behaviors may be different among mobile men or those in rural areas. Finally, the sample included men who were seeking VCT services and were HIV-negative. The results may not be generalizable to other populations such as HIV-negative men recruited from other settings, HIV-positive men, or women.

Many factors limit alcohol screening in both clinical and community-based settings, including social acceptability regarding alcohol use, limited provider time, and lack of training on alcohol screening and interventions [37, 38]. However, given that alcohol use, especially harmful or hazardous, is associated with high-risk sexual behaviors and negative health and social outcomes, it is important for providers in both settings to routinely screen patients for current alcohol use and provide alcohol reduction counselling to those who report harmful or hazardous drinking. Policy and structural-level changes regarding alcohol venues and sales in Namibia also may help to curb high-risk drinking. However, further research and guidance are needed to help inform these high-level changes.

## Conclusions

The findings indicate the need to re-evaluate HIV risk-reduction strategies, targeting alcohol use and unsafe sexual behaviors among HIV-negative men. Despite a high number of participants stating they had been exposed to HIV educational programs, sexual risk behaviors remained high. Approximately 33 % of respondents stated their health care providers had talked to them about their sexual behavior, alcohol use, or the connection between alcohol use and increased risky sexual behavior. While this study did not interview health care providers to verify these numbers, this is a strikingly low proportion of men at high risk for both HIV acquisition and alcohol use disorders that can recall harm reduction measures in health care settings. These results indicate the need for additional training to increase the frequency and skills for these discussions and to improve the method and types of HIV and alcohol-related educational interventions that are being implemented in healthcare and other settings.

## Abbreviations

BMI: Brief Motivational Intervention
CDC: Centers for Disease Control and Prevention
MoHSS: Namibian Ministry of Health and Social Services
PEPFAR: President’s Emergency Plan for AIDS Relief
STIs: sexually transmitted infections
VCT: voluntary HIV counseling and testing
WHO: World Health Organization
